# Evaluation of circulating serum cathelicidin levels as a potential biomarker to discriminate between active and latent tuberculosis in Uganda

**DOI:** 10.1371/journal.pone.0272788

**Published:** 2022-08-26

**Authors:** Ester Lilian Acen, David Patrick Kateete, William Worodria, Ronald Olum, Moses L. Joloba, Mudarshiru Bbuye, Irene Andia Biraro

**Affiliations:** 1 Department of Physiology, School of Biomedical Sciences, College of Health Sciences, Makerere University, Kampala, Uganda; 2 Department of Immunology and Molecular Biology, School of Biomedical Sciences, College of Health Sciences, Makerere University, Kampala, Uganda; 3 Pulmonary Division, Department of Medicine, Mulago National Referral Hospital, Kampala, Uganda; 4 Department of Medicine, School of Medicine, College of Health Sciences Unit, Makerere University, Kampala, Uganda; 5 Makerere Lung Institute College of Health Sciences, Makerere University, Kampala, Uganda; 6 Medical Research Council/Uganda Virus Research Institute and London School of Hygiene and Tropical Medicine Uganda Research Unit, Entebbe, Uganda; Fundació Institut d’Investigació en Ciències de la Salut Germans Trias i Pujol, Universitat Autònoma de Barcelona, SPAIN

## Abstract

**Background:**

Tuberculosis remains a major public health problem worldwide accounting for 1.4 million deaths annually. LL-37 is an effector molecule involved in immunity with both antimicrobial and immunomodulatory properties. The purpose of this study was to compare LL-37 circulatory levels among participants with active and latent tuberculosis and to determine its ability to discriminate between the two infectious states.

**Methods:**

A cross-sectional study was performed among 56 active tuberculosis patients, 49 latent tuberculosis individuals, and 43 individuals without tuberculosis infection. The enzyme-linked immunosorbent assay was used to assess LL-37 levels. Data analysis was performed using STATA software and Graph pad Prism version 8. Mann-Whitney U test was used for correlation between variables with two categories and the Kruskal-Wallis test for three or more categories.

**Results:**

The study had more female participants than males, with similar median ages across the three groups, 29.5, 25.0, and 23.0 years respectively. Active tuberculosis patients had significantly higher LL-37 levels compared to those with latent tuberculosis and without tuberculosis. The median/interquartile ranges were 318.8 ng/ml (157.9–547.1), 242.2 ng/ml (136.2–579.3), 170.9 ng/ml (129.3–228.3); p = 0.002 respectively. Higher LL-37 was found in the male participant with median/interquartile range, 424.8 ng/ml (226.2–666.8) compared to the females 237.7 ng/ml (129.6–466.6); p = 0.045. LL-37 had better discriminatory potential between active tuberculosis and no tuberculosis (AUC = 0.71, sensitivity 71.4% specificity = 69.8%) than with latent tuberculosis (AUC = 0.55, sensitivity = 71.4%, specificity = 44.9%). There was moderate differentiation between latent tuberculosis and no tuberculosis (AUC = 0.63, sensitivity = 44.9% specificity = 90.7%).

**Conclusion:**

Significantly higher LL-37 levels were observed among active tuberculosis patients than those without tuberculosis infection and were, therefore able to discriminate between active tuberculosis and other tuberculosis infectious states, especially with no tuberculosis. Further assessment of this biomarker as a screening tool to exclude tuberculosis is required.

## Introduction

Tuberculosis (TB) remains a major public health problem worldwide and accounts for about 1.4 million deaths, according to the World Health Organization (WHO) global report of 2019 [1–3]. Nonetheless, only about 5–10% of individuals succumb to the disease and a larger population remains with latent TB infection (LTBI), hence a great pool for disease progression [4]. Vitamin D deficiency is among the known nutritional risk factors that may predispose an individual to infectious diseases including TB [5]. Moreover according to studies progression of disease from LTBI to active disease may be caused by low body mass index (BMI) [6]. The equilibrium between the host immune system and *Mycobacterium tuberculosis* (Mtb) determines disease progression and is therefore important in the path physiology of TB disease [7, 8]. The intracellular Mtb acquires survival mechanisms in the host macrophages that cause complications in total clearance of TB infection. Conversely, diagnostic tools that might differentiate between LTBI and active TB disease are still deficient to date. Equally, our understanding of the protective mechanisms of the disease is unclear and more so the knowledge of the immune response to Mtb infection is still inadequate [9]. Therefore, improved knowledge of the host immune response to Mtb is important as it could inform the development of new biomarkers, especially those that can differentiate between active and LTBI [1, 10].

The 18kD human cathelicidin antimicrobial peptide (CAMP) called Leucine- Leucine 37(LL-37) is the only human host defense peptide. It is an effector molecule involved in the innate immune system with both antimicrobial and immunomodulatory properties causing broad-spectrum properties [11, 12]. In healthy mucosal surfaces and body fluids, there are minimal or no detectable LL-37 levels ranging between 2 to 5μg/ml. However, during infection or injury, there is an increase at sites of infection to about 30 μg/ ml [12–14]. LL-37 is secreted into the bloodstream by the bone marrow myeloid cells and these levels can be measured in plasma, serum, and saliva and the concentration is indicative of the bacterial load [15, 16]. The cationic end of LL-37 is responsible for the direct killing of microbes through its antimicrobial activity by interaction and disruption of its anionic cell walls and membrane and a highly conserved N- terminal with chemotaxis and oligomerization properties [14, 15, 17–23]. On the other hand, the proinflammatory activities of LL-37 involve the regulation of cytokines in TB infection. According to Dong-Min and Eun-Kyeong [24], Th1 cytokine Interferon-gamma (IFN-γ) up-regulates the induction of LL-37 by TLR2/1 while Th2 cytokine Interleukin 14 (IL-4) down-regulates it. Similarly, it is well established that interleukin 17 (IL-17) cytokines induce the production of antimicrobial peptides like LL-37, and that Mtb alters the function of IL-17 in primary TB infection [25].

Previous studies have reported variable LL-37 levels in TB infection and disease. Some reports account for elevated levels compared to their controls [5, 8, 26–28], while others have reported low levels in TB patients compared to controls [29]. In their studies, no difference has been found in both groups [1]. Therefore the variation in LL-37 levels among pulmonary TB (PTB) patients, LTBI Individuals, and those without TB infection is not well understood. We hypothesized that LL-37 levels are highly expressed in TB disease, and therefore have the potential of differentiating between active and LTBI. We aimed to assess the concentration levels of LL-37 among active TB patients compared to LTBI and those without TB infection and to determine the ability of LL-37 to differentiate between active and LTBI infection.

## Materials and methods

### Sample size, study design, and study population

This was a cross-sectional study including 3 groups; newly diagnosed active TB patients, LTBI individuals, and those without TB infection. Treatment naïve TB patients with positive Xpert MTB/Rif results aged between 15–65 years at the outpatient department of Kiruddu Hospital between the periods of July 2019 to August 2020 were consecutively enrolled in the study. All participants were screened for blood glucose levels and HIV serostatus. Participants with diabetes or hyperglycemia were excluded from the study to avoid possible confounders due to malabsorption of micronutrients. For the LTBI and the no TB groups, we selected stored samples from the KampalaTB (KTB) cohort study of household contacts based on QuantiFERON (QFT) and tuberculin skin test (TST) positive results. Individuals with (QFN^+^TST^+)^ were the latent TB and the (QFN^-^TST^-^) as the no TB infection group. Details of this study have been reported elsewhere [30]. After informed consent, a questionnaire was administered to the participants, and social demographic and clinical data were obtained.

### Measurement of serum LL-37 levels

LL-37 levels were performed in the Immunology laboratory at the College of Health Sciences, Makerere University. Five milliliters of venous blood samples were collected in plain vacutainer tubes and then centrifuged at 1000 g to obtain the serum which was stored at 80°c before analysis. The reagents were equilibrated to room temperature for 1 hour and samples were thawed. A 96 well Human (CAMP) competitive (ELISA) assay kit, catalogue abx150919 (abbexa Ltd, Cambridge, UK) was used to assess LL-37 levels according to the manufacturer’s instructions. Standards and control zero were diluted and 50 μl were added in duplicates to wells on the plate. The same volume of samples was then added to the remaining wells. A detection biotin-conjugated reagent A working solution was then added to the same wells and the plate was incubated at 37°C for 1 hr. The solution was discarded and the plate was washed 3 times using a prepared wash buffer. Another detection reagent B, horseradish peroxidase-conjugated streptavidin was added and a second incubation was done at 37°c for 30 minutes. The solution was once again discarded and the plate was washed three times. The 3,3’,5,5’-Tetramethylbenzidine (TMB) substrate was then aliquoted into each well and mixed thoroughly and the plate was incubated at 37°C for 10 min. A blue-colored product was formed which turned to yellow when a stop solution was added. The intensity of the color was measured spectrophotometrically at an optical density of 450 nm in a microplate reader. A standard curve (S1 File) was generated and the concentration of LL-37 in the unknown samples was calculated by plotting the optical density (ODs) against the concentration of standards in ng/ml. The calculated standard curve R2 was 0.98. The minimum detective range of the assay was between 123.5 ng/ml—10000 ng/ml. The intra-assays CV was < 10% and the inter-assay CV was < 12% with sensitivity of < 47.4 ng/ml.

### Measurement of total 25-hydroxyvitamin D_3_

Vitamin D3 was determined by the electrochemiluminescence binding assay with the COBAS 6000 immunoassay analyzer. Details of the methods of this analysis are reported elsewhere [31].

### Measurement of BMI and biochemical parameters

Anthropometric measurements of height and weight were measured among LTBI and those without TB infection. The BMI status was defined according to the American Heart Association which defines a normal BMI to lie between 18.5 to 24.9 kg/m^2^. Following the manufacturer’s instructions, biochemical parameters were measured. These tests included C reactive protein, (CRP) serum glutamic pyruvic transaminase, (SGPT), serum glutamic-oxaloacetic transaminase, (ALT/SGOT), Alkaline phosphatase (ALP), Albumin (ALB), Gamma-glutamyl transferase (GGT), protein (PRO) and creatinine (Cr) were performed using the Erba XL 640 Chemistry Analyzer.

### Statistical analysis

Data were analyzed using STATA software (Stata Corp. STATA version 16.0, College Station, Texas, USA, and Graph pad Prism (version 8). Participants with missing data were eliminated from the analysis. Normality of distribution was tested using Shapiro-Wilk, Anderson-Darling, D’Agostino & Pearson test, and Kolmogorov-Smirnov tests. Continuous data were summarized into medians and interquartile range (IQR), with a confidence interval (CI) at 95%, and an alpha of p<0.05 was considered significant at a power of 80%. Categorical variables were summarized as n (%). The Mann-Whitney U test was used for the analysis of two categories of variables while the Kruskal-Wallis test was used for three or more categories of variables. Correlation between LL-37 levels and the three study groups was performed using Shapiro-Wilk’s Test. A sensitivity test was performed using Dunn’s multiple comparison tests. Outliers were eliminated using the robust regression and outlier removal, in Graph Pad with Q at 1%. Linear regression analysis was performed to determine the association between LL-37 and other variables. The correlation between LL-37 and other variables was performed using Spearman’s Pair-wise correlation test. Receivers operating characteristics (ROC) analysis was performed to determine the utility of LL-37 as a diagnostic biomarker for TB differentiation. For accuracy, the area under the curve (AUC) was determined at 95% CI, and the sensitivity and specificity were determined.

### Ethical approvals

Ethical approvals were sought from Makerere University School of Biomedical Sciences Research and Committee (SOBSREC), reference number (#SBS-637), and National Council for Science and Technology reference number (HS2639). Waiver of consent was sought to use the KTB samples from (SOBSREC). Written informed consent was obtained from the active TB patients. Similarly, consent of the household contacts was obtained by the KTB study, and parents or guardians consented on behalf of the minors. The approvals and consent information of the KTB study are detailed elsewhere [30]. Patients’ personal information was kept confidential by using serial codes and no names were recorded on the questionnaire

## Results

### Baseline characteristics of study participants

A total of 148 participants consisting of 56 (37.8%) newly diagnosed active TB patients, 49 (33.1%) LTBI individuals, and 43 (29.1%) without TB infection were included in the study. Table 1 shows the baseline characteristics of the study participants. The median and interquartile ranges (IQR) of the ages of the participants were 29.5 (23.0–35.5) for active TB patients, 25.0 years (19.0–33.0) for LTBI, and 23.0 (13.0–32.0) years for those without TB infection (Table 1). The overall median age across the three groups was 26 years with an IQR range of (19–33) years, depicting a young population. The study predominantly had more female participants 91 (61.5%) than males. Of the 148 participants, 15.7% were HIV positive and according to the odds ratios analysis shown in Table 1, there was an association observed between HIV and TB status, especially between No TB and active TB states. The proportion of those who consumed alcohol was predominantly found in the LTBI and individuals without TB infection compared to the active TB patients. The majority of participants had BCG vaccination owing to the predominant presence of BCG scars observed. This variable also had an association with TB status with statistical significance between no TB and active TB, OR 6.6 (P< 0.001). Details of the associations of other variables with TB status are shown in Table 1. The baseline and clinical characteristics of LTBI individuals and those without TB individuals have been described elsewhere [30]. The median glucose levels of the active TB patients were 5.6 mmol/l. The majority of the active TB patients presented with a cough for over two weeks. Approximately 48% of them had performed x-rays on their first visit to the TB clinic. However, we did not record details of the abnormality diagnosed. Table 2 shows the details of the clinical factors. We used a reference range of 0–1.0 mg/dl to estimate serum CRP and found a mean of 7.7 mg/dl. Other biochemical tests were within the reference ranges and Table 3 has the details.

**Table 1:** Showing baseline characteristics of active TB patients’ latent TB individuals and those without TB infection.

Variables	No TB (N = 43)	LTBI (N = 49)	Active TB (N = 56)	No TB vs LTBI		No TB vs Active TB		LTBI vs Active TB	
	n (%)	n (%)	n (%)	OR (95%CI)	P	OR (95%CI)	P	OR (95%CI)	P
**Age (Median, IQR)**	23 (13–32.)	25 (19–33.)	29.5 (23–35.5)	1.0 (1.0–1.1)	0.097	1.05 (1.01–1.09)	0.010	1.01 (0.98–1.04)	0.472
**Sex**									
Male	17 (39.5)	15 (30.6)	25 (44.6)	Reference		Reference		Reference	
Female	26 (60.5)	34 (69.4)	31 (55.4)	1.5 (0.6–3.5)	0.371	0.8 (0.4–1.8)	0.610	0.5 (0.2–1.2)	0.142
**Alcohol**									
Yes	15 (38.5)	15 (31.9)	11 (20.8)	0.8 (0.3–1.8)	0.526	0.4 (0.2–1.0)	0.046	0.5 (0.2–1.3)	0.156
No	24 (61.5)	32 (68.1)	42 (79.3)	Reference		Reference		Reference	
**Smoking**									
Yes	5 (12.8)	9 (19.1)	8 (15.1)	1.6 (0.5–5.3)	0.431	1.1 (0.3–3.8)	0.838	0.7 (0.2–2.0)	0.509
No	34 (87.2)	38 (80.9)	45 (84.9)	Reference		Reference		Reference	
**BCG scar**									
Yes	32 (82.0)	29 (61.7)	24 (46.2)	Reference		Reference		Reference	
No	7 (18.0)	18 (38.3)	28 (53.8)	2.8 (1.0–7.8)	0.042	6.6 (2.5–17.4)	<0.001	2.3 (1.0–5.1)	0.038
**HIV Status**									
Positive	2 (4.7)	4 (8.2)	17 (30.9)	1.8 (0.3–10.5)	0.501	9.2 (2.0–42.4)	0.005	Reference	
Negative	41 (95.3)	45 (91.8)	38 (69.1)	Reference		Reference		5.0 (1.6–16.2)	0.007

No TB without infection is the control group, LTBI is the latent TB group and Active TB are pulmonary TB patients. n is several variables with their percentage. Age is in median and IQR (interquartile range) P values < 0.05 is significant. OR > 1 have an association

**Table 2:** Showing the clinical characteristics of the active TB patients.

Clinical characteristics	n	%
Chest Xray *(n = 56)*		
No	29	51.7
Yes	27	48.2
Cough *(n = 56)*		
No	1	1.9
Yes	55	98.2
Other drug use (n = 53)		
No	45	84.1
Yes	8	15.1
Genexpert positive (n = 56)		
No	0	0
Yes	56	100
Rifampin resistance (n = 56)		
No	52	92.9
Yes	4	7.1

For the x ray No means the patient did not have one.

**Table 3:** Showing the biochemical parameters the active TB patients.

Characteristics	Mean (SD) /units
CRP	7.7(6.2) mg/dl
SGPT	5.4(4.9) U/l
SGOT	14.5(15.1) U/l
ALP	60.0(29.8)) g/dl
ALB	5.9(12.1) g/dl
GGT	39.8(31.4) U/l
CREA	= 7.7±6.2 mgdl
PRO	7.6(1.1)) g/dl

### Serum LL-37 levels among active TB patients, latent TB individuals, and those without TB infection

**Fig 1:**
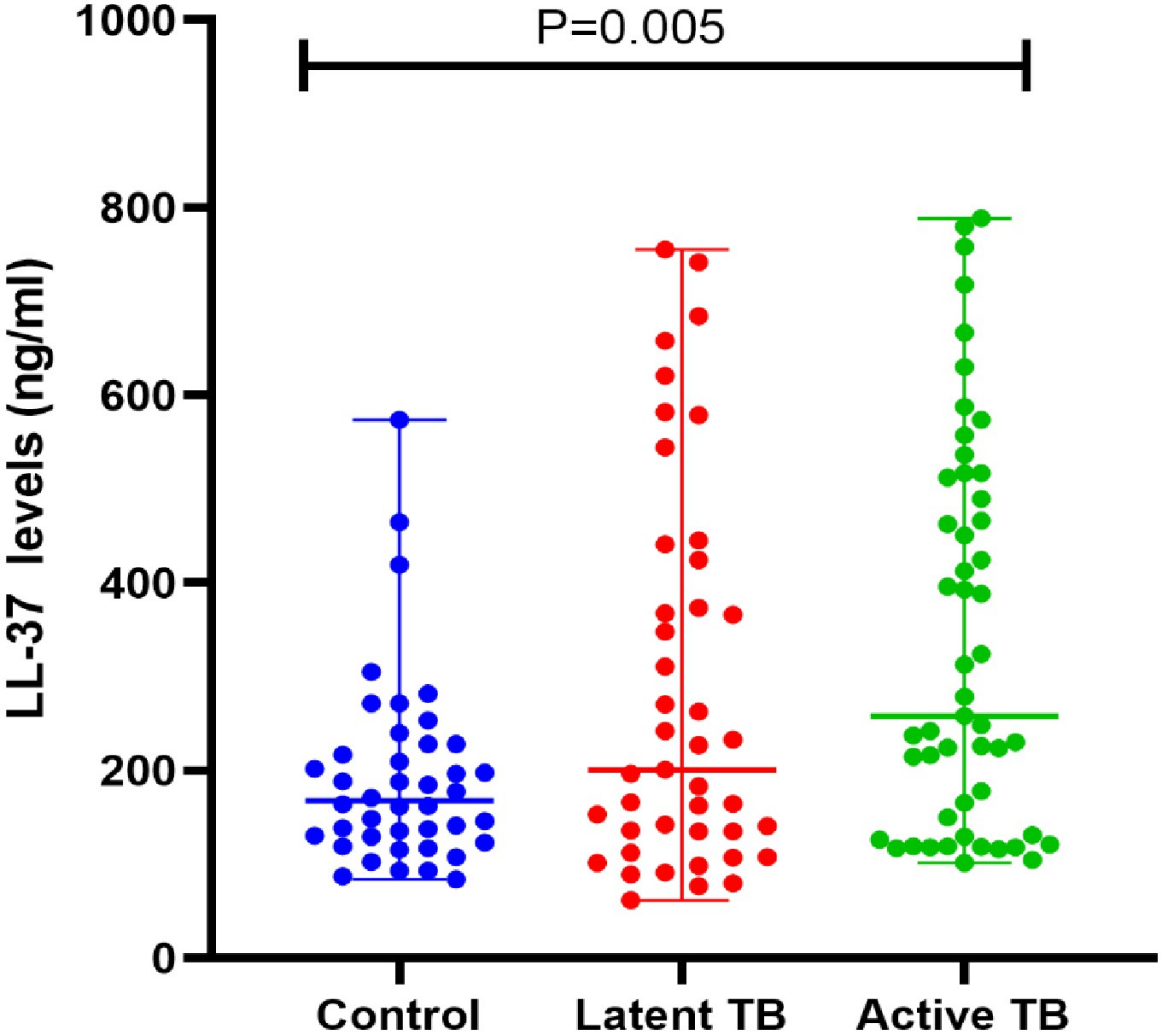
Distribution of serum LL-37 levels among active TB patients, LTBI individuals, and those without TB infection. Mann-Whitney; p = 0 0008 Active TB vs control, p = 0.1430 Active TB vs LTBI, p = 0.1374 LTBI vs control. N = 136. Each dot represents an individual. Median serum concentrations were significantly higher among the active TB patients compared to the latent and non TB individuals. p values are performed by Mann -Whitney test.

The normality of data was assessed and a Gaussian distribution was not realized at P<0.0001. However, no log transformations were performed therefore non-parametric tests were used for further analysis of data. A further analysis was carried out to remove outliers and consequently we removed 12 outliers reducing our sample size from 148 to 136 participants consisting of 51 active TB patients, 43 LTBI individuals, and 42 of those without TB infection. The median LL-37 concentration levels among the study groups were 318.8 ng/ml with an IQR (157.9–547.1) for active TB, 242.2 (136.2 + 579.3) LTBI, and 170.9 (129.3–228.3) for those without TB infection(p = 0.0005), (Table 4). The highest and lowest LL-37 levels reported were1480. 6 ng/ml and 61.0 ng/ml respectively. When LL-37 levels were analyzed across various risk factors in the three groups, we found significantly higher LL-37 levels in the smokers with active TB and LTBI, P = 0.011 and0.017 respectively compared to non-smokers. Other risk factors did not show any statistically significant difference (Table 4). A post hoc analysis using Dunn’s multiple comparisons tests was further performed and a strong statistical difference was detected between the active TB patient and those without TB infection groups (p = 0.002), and a borderline difference between the LTBI study participants and those without TB infection(p = 0.058), and a weak difference between active TB and latent TB, p = 0.8118 (Fig 1). Further analyses of LL-37 levels were assessed among the different variables as detailed in Table 4. LL-37 levels were higher in the male participants compared to females with a statistical significance of p = 0.045 for the active TB patients, while in the other two groups it was higher among the females compared to the males with a marginal difference among those with no TB infection, p = 0.051.

**Table 4:** Bivariate analysis of LL-37 levels and categorical variables in active TB patients, Latent TB individuals, and those without TB infection.

LL-37 levels (ng/ml)	No TB (N = 43)	P	LTBI (N = 49)	P	Active TB (N = 56)	P
	Median (IQR)	value	Median (IQR)	value	Median (IQR)	value
**Overall**	170.9 (129.3–228.3)		242.2 (136.2 + 579.3)		318.8 (157.9–547.1)	
**Age (Rho, P)**	-0.104^a^	0.507	-0.247^a^	0.087	0.045^a^	0.743
**Sex**						
Female	146.1 (117.0–188.1)	0.051	196.8 (107.7–424.8)	0.618	237.7 (129.6–466.6)	0.045
Male	192.7 (137.5–271.5)		256.4 (136.2–582.1)		424.8 (226.2–666.8)	
**Alcohol intake**						
Yes	138.8 (117.0–228.3)	0.386	310.7 (140.8–658.0)	0.553	243.1 (131.1–517.2)	0.268
No	163.1 (133.0–240.8)		229.7 (110.1–512.1)		258.3 (216.1–587.8)	
**Smoking**						
Yes	148.7 (135.5–253.3)	0.817	741.9 (310.7–941.3)	0.017	524.7 (410.5–748.6)	0.011
No	163.1 (123.5–228.3)		190.0 (134.9–441.2)		237.7 (129.6–466.6)	
**BCG scar**						
Yes	161.9 (126.4–234.1)	0.869	262.6 (162.6–620.8)	0.220	388.3 (121.0–517.2)	0.775
No	164.3 (117.0–228.3)		168.2 (107.7–445.0)		258.3 (216.1–587.8)	
**HIV Status**						
Positive	151.5 (138.8–164.3)	0.645	237.4 (165.4–591.7)	0.971	226.2 (150.2–466.6)	0.434
Negative	177.5 (129.3–228.3)		262.6 (136.2–579.3)		356.4 (165.6–573.7)	

^a^ Pairwise Correlation Rho; Mann-Whitney U test was used for the rest of the comparisons.

IQR = interquartile range represents the 25^th^ and 75^th^n percentile

### Serum LL-37 levels by gender and HIV infection status

**Fig 2:**
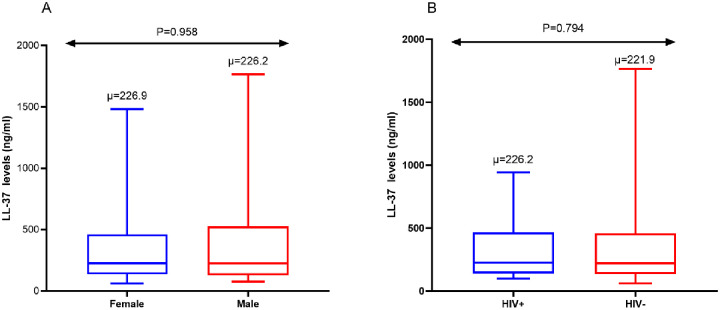
Overall median levels of LL-37 by gender and B. HIV infection status among all three groups combined. Mann-Whitney U tests were performed. The upper and lower end of the box represents the 75^th^ and 25^th^ percentile.

When LL-37 levels were analyzed according to gender and HIV (Fig 2), no statistical significance was noted. However, when the analysis was performed by individual groups we noted a marginally significant p-value by gender among the active TB patients, p = 0.065. Analysis between the other groups did not show a significant difference; LTBI, p = 0.505, and control, p = 0.074. A Sub-analysis using Spearman’s correlation was performed between LL-37 and HIV, age, and sex no association was found between LL-37 levels and age (rho: -0.023, P = 0.783), sex (P = 0.958), or HIV status (P = 0.794). When a post hoc sensitivity analysis was performed between LL-37 levels and HIV the significance was found among active TB patients and those without TB infection, P = 0.004.

### Association of LL-37 and TB related risk factors among active TB patients

A linear regression analysis was performed against some predictor variables and LL-37 levels among the active TB patients. We found a strong association between LL-37 and male gender, p = 0.020, and smoking, p = 0.027, (Table 5). Noteworthy however was a very weak negative association between LL-37 and CRP levels with no statistical significance observed, r = -0.03, (P = 0.84).

**Table 5:** Linear regression analysis of predictor variables and LL-37 levels among active TB Patients.

Variable	Unadjusted Coefficient	95% Confidence Intervals	P value
**Age**	0.4	-9.4 to 10.3	0.927
**Sex**			
Female	Ref.		
Male	224.3	37.3 to 411.2	0.020
**BCG scar**			
No	Ref.		
Yes	-62.4	-270.1 to 145.4	0.549
**Alcohol**			
No	Ref.		
Yes	135.4	-116.6 to 387.4	0.286
**Smoking**			
No	Ref.		
Yes	311.0	35.9 to 586.2	0.027
**HIV**			
No	Ref.		
Yes	-130.8	-342.5 to 80.9	0.221

Smaller values made as references p value < 0.05

### Correlation of LL-37 and cytokines levels in LTBI and those without TB infection

A pair-wise correlation was performed between LL-37 and some cytokines among LTBI and those without TB infection. There was a weak positive correlation between LL-37 and IFNλ0 among those without TB infection with a statistical significance, (Table 6). Among the LTBI there were weak associations between LL-37 and IL17A0 and IL17A1 with no statistical significance. Furthermore weak associations between LL-37 levels and other cytokines were observed with no statistical significance.

**Table 6:** Correlation between LL-37 and cytokines in those without TB infection and LTBI.

Cytokines	No TB infection		LTBI	
	Rho (ρ)	P	Rho (ρ)	P
TNFα0	0.009	0.960	0.153	0.353
IFNλ0	0.386	0.022	-0.086	0.602
IL17A0	-0.150	0.413	-0.226	0.198
IL17F0	-0.078	0.666	0.016	0.927
IFNλ1	0.048	0.784	0.263	0.106
TNFα1	0.147	0.401	0.244	0.135
IL17A1	-0.065	0.729	-0.256	0.145
IL17F1	0.003	0.986	0.099	0.577

Spearman’s Pair-wise, TNF tumor necrosis factor, IL interleukin, IF interferon

P = 0.05 is significant

### Correlation of LL-37 and BMI among LTBI and those without TB infection

The median BM1 of the LTBI was 22(20–27) and the BMI of those without TB infection was 22.5(20–25). When a correlation analysis was performed between LL-37 and BMI a very weak negative association with no significance was observed, r = -0.18 (p = 0.19).

### Correlation of serum 25-hydroxyvitamin D and LL-37 levels among active TB patients LTBI and those without TB infection

**Fig 3:**
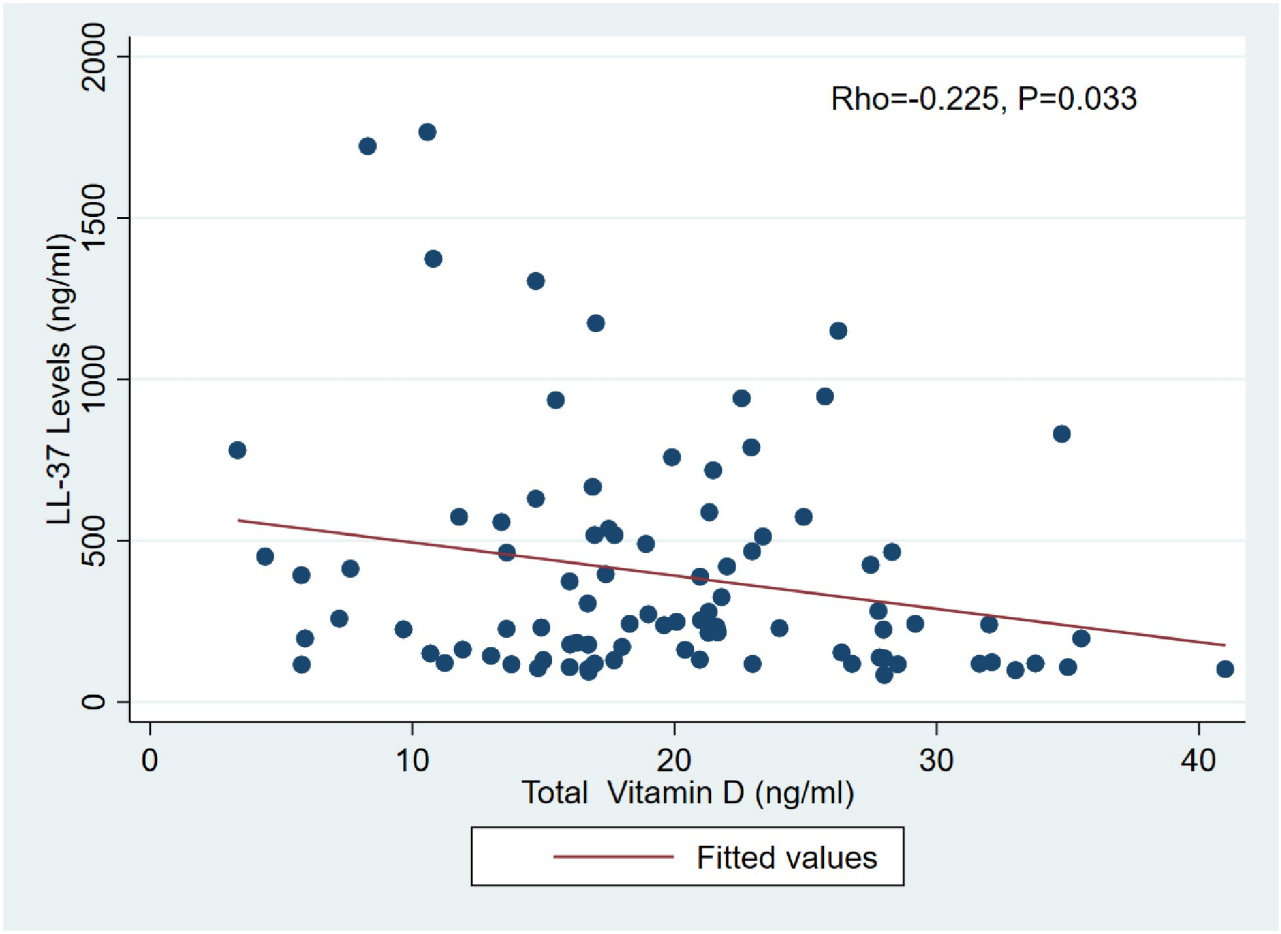
Correlation of LL-37 and total vitamin D levels in ATB patients, LTBI, and those without TB infection.

The median serum levels of the study population were18.3 ng/ml (13.7–23.7). A significant difference was observed in the three study groups, active TB patients 17.4 ng/ml, (2.6, 21.4) LTBI, 22.6 ng/ml (16, 29.2) and those without TB infection were 21.5 ng/ml, (916.7,27.8). A proportion of hypovitaminosis D, 85.3% were observed in the study population. Details of these findings have been reported elsewhere [31]. When a correlation analysis was performed between LL-37 and total 25-hydroxyvitamin D3, a statistically significant weak negative association was observed as shown in Fig 3.

### Assessing the potential use of LL-37 levels to discriminate between TB status

**Fig 4:**
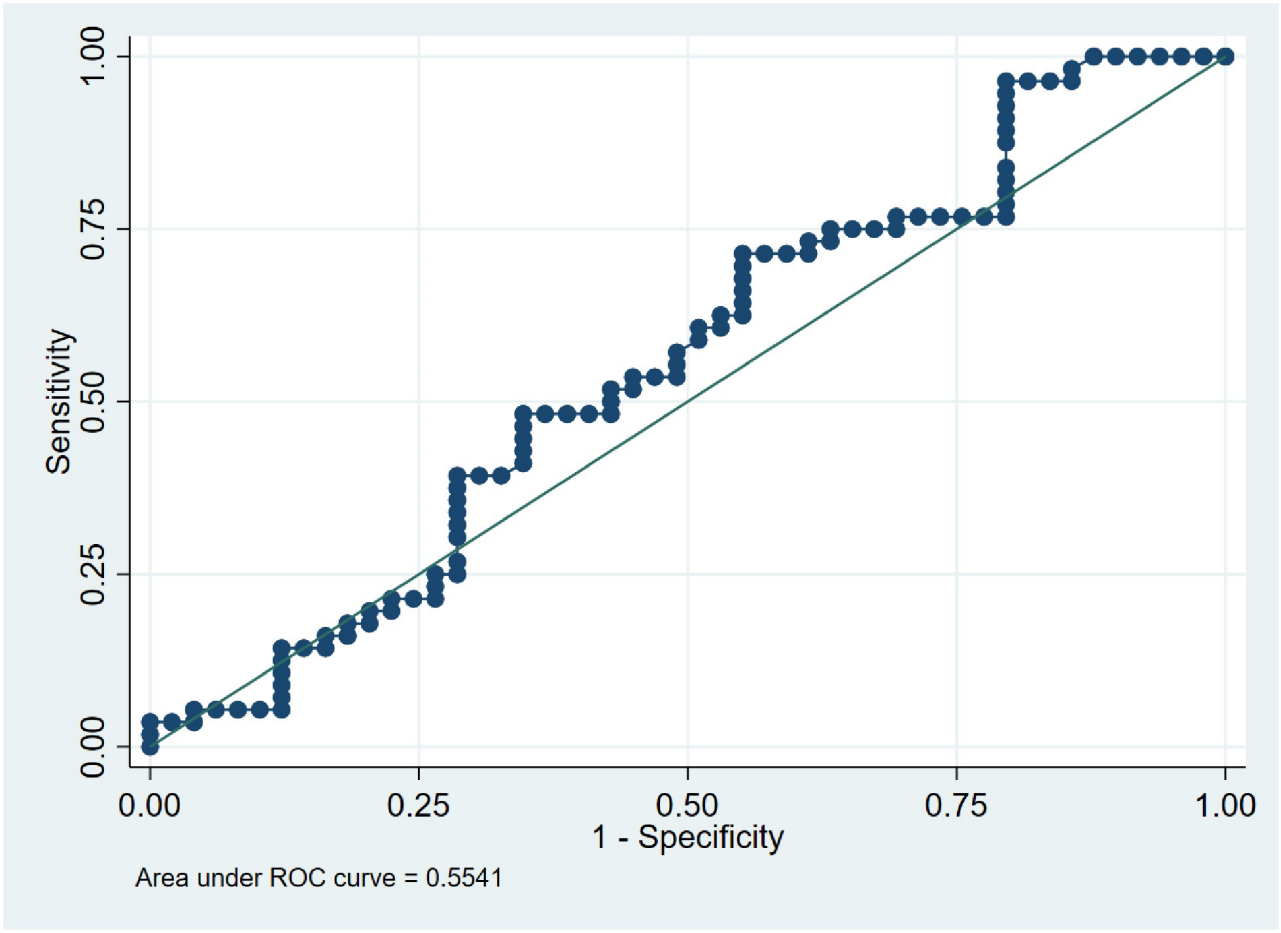
ROC curve showing an accuracy of LL-37 levels in differentiating between active TB disease and LTBI with a sensitivity of 71.4% and specificity of 44.9%.

**Fig 5:**
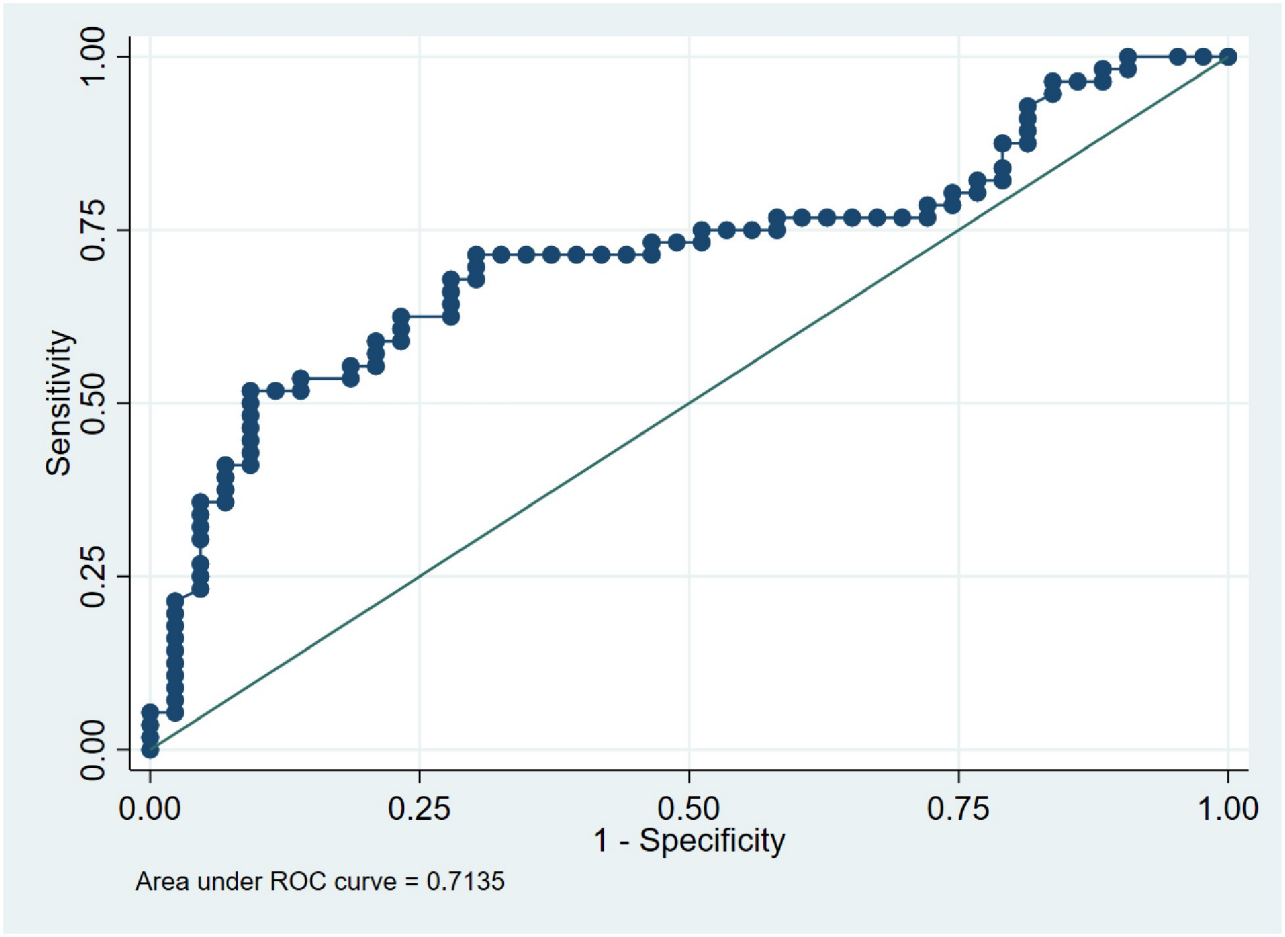
ROC curve showing an accuracy of LL-37 in differentiating between active TB and those without TB infection with a sensitivity of 71.4% and specificity of 69.8%.

**Fig 6:**
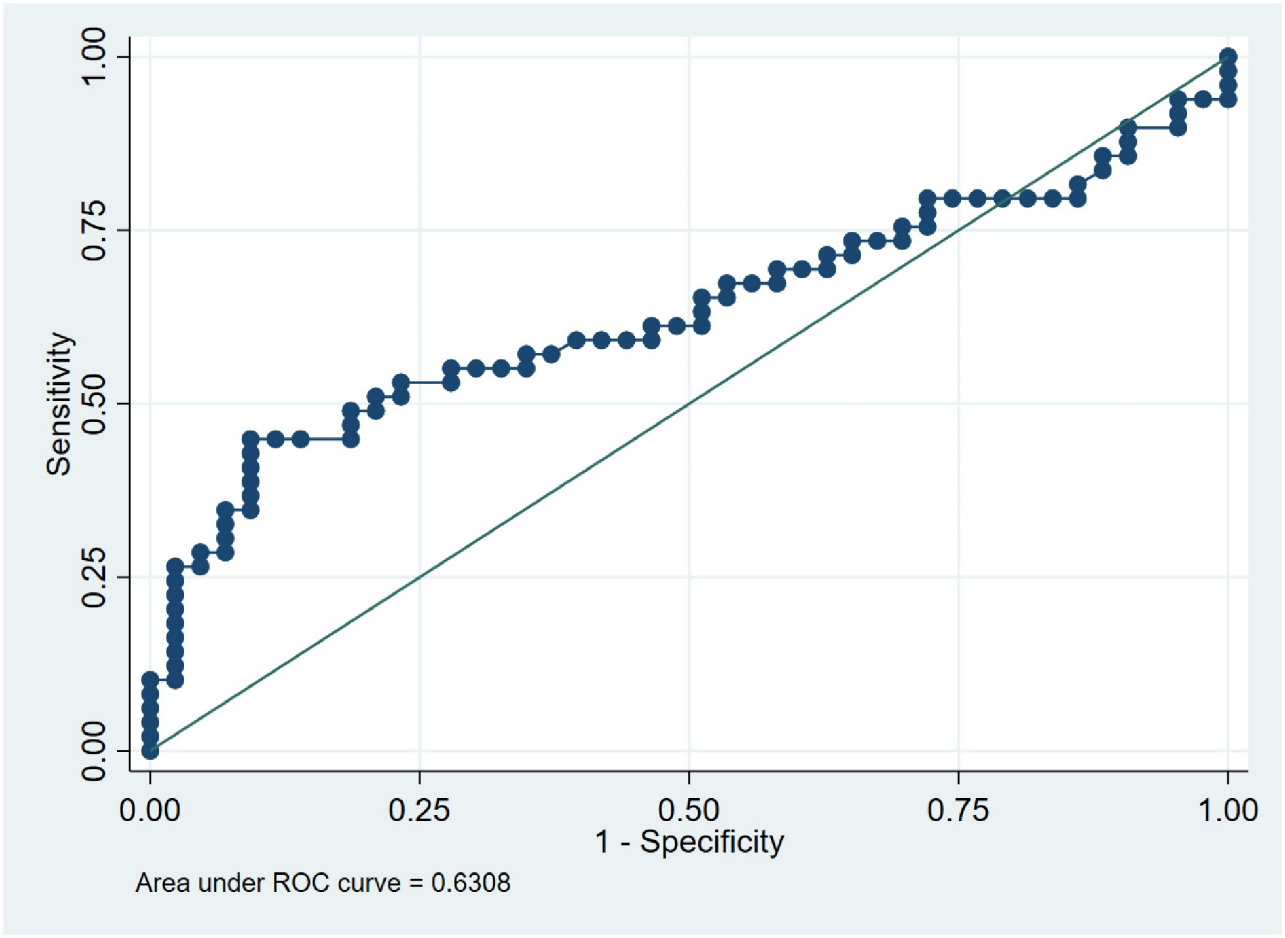
ROC curve showing an accuracy of LL-37 levels in differentiating between LTBI and those without TB infection with a sensitivity of 44.9% and specificity of 90.7%.

We performed ROC analyses to determine the utility of LL37 as a differential biomarker for TB infection. Analysis was performed against active TB and LTBI at a cut-off of > 214.8 ng/ml. The AUC was 0.55 (95% CI: 0.44–0.67), Fig 4. LL-37 correctly classified active TB disease at 59.1% with a sensitivity of 71.4% and a specificity of 44.9%. When analysis of active TB and those without TB was performed the AUC was 71.3 (95% CI: 0.61–0.82) as shown in Fig 5. LL-37 correctly classified active TB at 70.7% and sensitivity of 71.4% and specificity of 69.8% at a cut-off of 214.8 ng/ml. When the analysis was performed between LTBI and those without TB infection at a cuff of > 310.7 ng/ml, the AUC was 0.63. LL-37 correctly classified LTBI at 66.3% with a sensitivity of 44.9% and specificity of 90.7% as shown in Fig 6.

## Discussion

In this study, we found elevated circulatory LL-37 levels among active TB patients compared to those without TB infection. Experimental studies have suggested an elevation of LL-37 expression at the onset of TB disease, up to day 28, and a decrease at day 60 [32, 33]. This early elevation of LL-37 plays a role in the control of the detrimental effects of bacterial infection by inhibiting the secretion of TNF α. Our study recruited newly diagnosed TB patients who had not received therapy, therefore presume that they were not at an advanced progressive disease stage owing to similar findings of high LL-37 levels as opposed to the decreased levels as the disease advances due to high bacillary load and extensive tissue damage. Equally, findings from another study [34] among the same TB groups and controls found raised levels of the antimicrobial peptide in the progressive TB patients compared to the latent and controls. However, in the same study, they did not find a significant difference in LL-37 levels between the LTBI and control group. Furthermore, our findings are in concurrence with previous studies that reported high LL-37 levels among active TB patients [8, 26, 28, 35, 36]. There is an assumption that host defense peptides including human LL-37 function actively during early infection for elimination and containment of the disease. Therefore an effective immune response may determine the outcome of the disease [37]. This minor difference in LL-37 levels between active TB patients and LTBI may be due to a process of the latent infection progressing to active TB disease.

When LL-37 was examined by age, we found a minimal negative correlation between the two variables. This finding is similar to a study by Alvarez-Rodriguez *et al* (2012) who found a decrease in LL-37 with age [38]. However other studies have accounted differently that LL-37 levels increased with age [39, 40].

Regarding the comparison of LL-37 levels in HIV+ and HIV–individuals, among the three study groups no significant difference was observed between the active TB patients, LTBI individuals, and those without TB. A positive relationship was detected between the male gender and LL-37. Our finding is comparable to a study by Stukes *et al* (2016) who found high LL-37 levels with a positive association [41]. We equally found a positive relationship between smokers with LL-37 levels. Smoking is a known risk factor for TB infection and disease. We may assume that these two variables may be related because a majority of the smokers were male.

IFNɣ plays a role in the immunity of TB and is responsible for the polarization process. A previous in-vitro study demonstrated that LL-37 inhibits the immune response of IFNɣ [42]. This study found a statistically significant, weak positive correlation between LL-37 and IFNɣ0 among the individuals with no TB infection (r = 0.38, p 0.022). According to a previous report by Dong-Min and Eun-Kyeong, (2011), IFNɣ up-regulates LL-37 [24]. On the contrary negative correlation between LL-37 and IFNɣ was observed among PTB patients in a previous study by Zhan and Jiang (2014) [26]. The observed positive correlation in the control group may confirm the hypothesis that once alterations have occurred in LL-37 levels due to the presence of pathogens, then modifications will be made in its function. However, in the LTBI, a very weak negative correlation (r-0.086) was observed and it was not significant. Additionally Noteworthy was a weak negative association that was observed between LL-37and IL-17A0 among the LTBI. Although it was not statistically significant these results may provide an insight into the functional status of LL-37. IL-17 assists in the granulomas development and control of Mtb growth during early TB infection [7]. Although IL-17 induces the production of LL-37, it is altered by Mtb. The negative association could be due to the increased reduction of IL-17 as bacterial load increases and LL-37 is elevated. Based on these observations we may presume that the immunomodulatory relationship between LL-37 and cytokines may be dependent on TB status We did not perform cytokine analysis for the ATB patients and therefore were unable to examine the correlation between LL-37 and the IFNɣ as reported in earlier studies. However, based on the analysis performed, these could be preliminary mechanistic studies that may explain the function of LL-37 in the immunity of TB.

The analysis between LL-37 and CRP in the ATB patients revealed a very weak negative association, which was not significant. CRP is an acute inflammation marker and may be used to determine the severity of TB disease since it’s associated with elevated levels [43]. As CRP increases LL-37 levels decrease, this would have been being surprising since LL-37 expression determined by the bacterial load. Noteworthy is that CRP is also in the inflammatory process by activation of the classical complement leading to the protection against infection [44]. We, therefore, presume that as more protection is provided by CRP bacterial load is reduced and leading to a reduction in LL-37 levels.

As previously established low BMI plays a role in immunity and predisposes individuals to infections [6]. Further evidence from another study showed that low BMI was associated with TB mortality [45]. The BMI of our LTBI and those without TB infection was normal with a weak negative non-significant association with LL-37. This is contrary to a report from a study among LTBI and healthy controls that found a positive association between BMI and LL-37 and that individuals with low BMI had reduced LL-37 levels [6]. Interestingly unlike our study participants in that study were recruited from the community and not TB household contacts yet they still had a lower BMI.

Concerning vitamin D status majority of the participants had insufficient levels with an observation of severe deficiency as reported elsewhere [31]. Except for LL-37 expression in response to the presence of pathogens and injury a physiologically important metabolite from the vitamin D pathway, 1,25dihydroxivitamin D3 enhances the production of LL-37 [13]. Additionally, alternative pathways that could influence vitamin D status through the cytochrome P450 molecule have been suggested [46, 47]. Consequently, the knowledge of the vitamin D status of the study participants is an integral part of the study. Recent research advances have attributed another functional activity, the hypoxia-inducible transcriptional factor (HIF 1α) to LL-37. This path is an immune and inflammatory mediator of the hypoxia pathway in the cells of mammals including humans and is responsible for the reduction in LL-37 [48]. This pathway is an important aspect for consideration in the study of LL-37 expression. The present study found high LL-37 and low vitamin D levels among the ATB patients compared to other groups with a weak positive correlation. Previous studies have reported similar findings, and our recent systematic review pooled several such primary studies and reported high LL-37 levels and low vitamin D among PTB patients compared to the controls [49].

We performed an accuracy analysis test to determine the utility of serum LL-37 levels as a biomarker to discriminate LTBI from active disease by ROC analysis. In this test, a cutoff of > 214.8 ng/ml was selected and showed the AUC = 0.55 with high sensitivity and moderate specificity. This is an indication that the test had the minimal discriminating ability. This is in agreement with the Dunn’s comparison multiple sensitivity test we performed among the three groups and found, P = 0.81 a non-significant difference between active TB patients and LTBI. When we further analyzed if we could distinguish between active TB patients and those without TB infection there was a better discriminatory ability, AUC = 0.71, and correctly classified active TB patients at 70.7% with a minimum difference between sensitivity and specificity which turns out to be a good discrimination test. Once more our sensitivity test had found a significant difference between active TB and those without TB infection. These ROC analysis results are closer to one study that performed a ROC analysis to test for the discriminatory ability of LL-37 between pulmonary TB patients and healthy endemic controls and found the AUC of 0.78 to have a better discriminatory ability compared to other molecules [27]. The discrimination between LTBI and those without TB had minimal discriminating ability between the two groups. However, the sensitivity test showed a marginally significant difference as shown by the results. Our findings of the low discriminating ability between LTBI and those without TB infection may be supported by a previous study that suggested that it is likely that household contacts may have underlying latent TB infection [8]. On the contrary, another study did not find a difference between the two groups [34]. However, no ROC analysis was performed to determine the discriminatory ability of LL-37. Overall the ROC curve analysis of active and latent TB disease falling on the diagonal line with low AUC at 0.55 demonstrates low accuracy of the circulatory LL-37 levels in discriminating the two states of the disease. This may probably be explained by a hypothesis of previous research suggesting that TB should be viewed as a continuous spectrum [50]. A previous in-vitro study on the expression of LL-37 in different epithelial cells infected with Mtb showed that high levels of LL-37 were produced in 18hrs in a dose-dependent manner and low bacilli were detected in the alveoli macrophages at this time [10]. This demonstrates that LL-37 is effectively involved in epithelial lung primary infection. In the same study, LL-37 production was not detected in the tuberculosis granulomas, an indication that LL-37 is probably not involved in chronic infection. This study as earlier mentioned in reports [32, 33] indicates that LL-37 is important at the onset of infection. LL-37 is constitutively produced in the bloodstream and increases after infection and it should be noted that other pathways are involved in the up and down-regulation of LL-37 which may affect its concentration.

To our knowledge, this is the first study to perform a ROC analysis for the determination of LL-37 as a potential differential biomarker against active TB, LTBI, and those without TB infection. The study forms a basis for further research on the discriminatory ability of LL-37 between TB disease and no TB infection using the cutoffs provided. We provide preliminary mechanistic studies of LL-37 function by examining the association between LL-37 and cytokines. Our data confirm the importance of LL-37 in the immunity of TB disease and that the continuous spectrum of TB infection and disease may be leading to the low discriminatory ability of LL-37 between the LTBI and active TB disease. The strength of our study lies in the fact that our controls and LTBI were household contacts of active TB patients from a previous cohort.

This study did not perform an analysis of the hematological parameters, therefore, acknowledges this limitation. Secondly, anthropometric data of the active TB patient was not collected consequently were unable to measure their BMI. Furthermore, due to inadequate sample volume analysis of cytokines in the same group was not performed. The lack of known internationally accepted reference ranges for LL-37 levels may pose a limitation in the synthesis of the functional ability of this molecule. Variable LL-37 concentration levels have been reported in previous studies. Further still, these studies have had variable detection limits with variable assay ranges and units. We used an assay range of 123.5–10,000 ng/ml while other studies have used much lower assay ranges [7, 8, 27, 51, 52]. This poses complications in comparison of results from one study to another. There is still no gold standard to differentiate LTBI from active disease therefore our LTBI participants were diagnosed using TST and QTF tests which cannot differentiate between active TB and LTBI.

Careful study design and selection of study participants to include newly diagnosed active TB patients, those on treatment, the LTBI, and control groups should be paramount to target different phases of the infection. Environmental factors including the length of exposure should be considered when including household contacts. The inclusion of other healthy individuals from the population as another control group with no history of exposure may be informative in the discovery of LL-37 as a suitable diagnostic biomarker. It is important to note that LL-37 local tissue expression in TB infection could probably reveal pertinent information on its discrimination ability.

## Conclusion

Significantly higher LL-37 levels were observed among the active TB patients than those without tuberculosis infection indicating that elevated LL-37 levels are expressed in an active TB state. Therefore LL-37 was able to discriminate between active TB and other TB infectious states, especially with no TB. Further assessment of this biomarker as a screening tool to exclude TB is required.

## Supporting information

S1 File LL-37 standard curve. (XLSX)

S1 Appendix Questionnaire study tool. (DOCX)

S1 Dataset (ZIP)
