# Supplementary material for: Evaluation of circulating serum cathelicidin levels as a potential biomarker to discriminate between active and latent tuberculosis in Uganda
Source: PLoS One. 2022 Aug 26;17(8):e0272788. doi: 10.1371/journal.pone.0272788 (PMC9416991; doi:10.1371/journal.pone.0272788)
Supplement: S1 Appendix — (DOCX) [file pone.0272788.s002.docx]

# ENGLISH QUESTIONAIRE

**DATA COLLECTION TOOL FOR THE DETERMINATION OF VITAMIN D BINDING PROTEIN GENE POLYMORPHISM AND VITAMIN D BIOAVALBILTY ON CATHELICIDIN EXPRESSION IN TB INFECTION AND DISEASE AT KIRUDDU HOSPITAL**

Date…………………Study Number………………..………Tel no……………………..

**SOCIAL- DEMOGRAPHIC INFORMATION**

1. Age (years)………………..……….
2. Gender Male…………………..…….. Female……………………………
3. Religion: Protestant………………... Catholic………………..… Muslim………….……

Others (specify)…………..………………..……..…….

1. District of origin……………………….……………
2. Education level: O-level…………………………………. A- Level…………………………… Bachelors Degree…………………………………….….. Masters degree…………….……….

Uneducated…………………………………others (specify)……………..………………..

1. Occupation…………………………………….……
2. Tribe………………………………….……………..
3. Marital status: single……..…… Married/cohabiting……………………………

Divorced/separated……………………………………………………………………

1. Homeless ……………………………………………………………………………….
2. Living in a shelter with …………………………ventilation……………………………
3. Living in a single room……………………ventilation…………………………………
4. Any other………………………………………………………………………………

**ECONOMIC STATUS AND SUNSHINE EXPOSURE**

1. Working conditions

Outdoor conditions …………………………………….………………………………….

Inside though out…………………………………………………………………………..

Any crowding condition…………………………………………………………………………

1. Healthcare access…………………………………………………………………………
2. Food security……………………………………………………………………………..
3. Foods with dietary vitamin D …………………………………………………………….

**HEALTH KNOWLEDGE, ATTITUDE AND BEHAVOUIR**

1. Do you know the medical condition you are suffering from? Yes No

If yes, what is the condition called....................................................................................................

1. Do you know the likely cause of your condition? Yes No

If yes, what is the likely cause?

……………………………………………………………………………………………………… ………………………………………………………………………………………………………………………….………………….……………………………………………………………… Do you have history of contact of a Tuberculosis patient ………………………………………

If yes how many years ago…………………………………………………………………………

1. Illicit drug uses
2. Smoking…………………………………………………………………
3. Alcohol……………………………………………………………………………………
4. Any other drug …………………………………………………………………………

Clinical information

BCG scar: present…………………………. Not present……………………………………….

Diabetes mellitus…............................................................................................................................

Malignancy ………………………………………………………………………………………..

HIV status………………………………………………………………………………………….

Chest X-ray

Normal ......................................................................................................................................

Abnormal …………………………………….............................................................................

Coughing……………………………………………………………………………………………

Positive genexpert ………………………………………………………………………………..

**LABORATORY PARAMETERS**

Glucose levels........................................... mmol/l

Vitamin D levels……………………..…. ng/ml

Cathelicidin levels………………………ng/ml

Cytokine levels…………………………..pg/ml

Biochemical tests

**..................................................................................................................**

**..................................................................................................................**

**...................................................................................................................**

**..................................................................................................................**

**GC ISOFORMS**

*Gc1F* …………………………………………%

*Gc1S*…………………………………………%

*Gc 2*…………………………………………. %

**LUGANDA QUESTIONNAIRE**

OLUPAPULA LWE BIBUUZO

**EKYOKOZESA OKUNONNYEREZA BIKI EBILEETERA ENKYUKAKYUKA MU BUSIMU OBUKOZESEBWA VITAMIN D AWAMU N’EMBEERA YA VITAMIN D MU BALWADDE BA KAFUBA MU DDWALIRO LYE MULAGO.**

Ennaku z’omwezi……………….…Ennamba y’omusomo……………Essimu ……………….

**EBIKUKWATAKO**

1. Emyaka……………….………………..………
2. Ekikula: Musajja………………..…….. Mukaszi……………………………
3. Eddiini: Mukurisitayo………………......... Mukatuliki…………………………… Musiraamu…………………………….……

Endala (Nyonyola)……………………………………………………………..…..

1. District gye bakuzaala……………….……………..
2. Okusoma kwo: Sekendule (1-4) ……………………………

Sekendule (5-6)…………………………… Diguli esooka…………..…………………. Diguli eyo kubiri……….………….………. Sasoma ko………...………………….… Ebirara ( Nyonyola)…………………

1. Omulimu……………………………………………………………..…………….
2. Eggwanga lyo…………………………………………….………….……………..
3. Ebyobufumbo: Muwuulu……..………………… Mufumbo……..……………

Mwayawukana…………………………………………………………………..

1. Tolina wo beela ……………………………………………….
2. Osula mu nnyumba?.......................... Eliko obumooli na madirisa?........................
3. Ennyumba gy’obeera mu ya busenge bimeka?………… biliko obumooli na madirisa?............................
4. Ebirara byona………………………………………..……………………………

**EKIKWATAGANA KU BYENFUNA**

1. Embeera gy’okoleramu elye tya?………………………………………..…………………

Okolera wabweru………………………………………………………………………………

Oba mundda…………………………………………………………………………………..

1. Waliyo omugatiko gwa abantu?…………………………………….………………………
2. Waliwo we mufunira obujjanjabi?…………………………….……………………………
3. Waliwo ebyokulya ebyo mugaso mubungi?………………………………………………..
4. Waliwo ebyokulya omuli vitamini D………………………………………………………

**OKUMANNYA EBY’OBULAMU NE NEYIISA**

1. Omanyi obulwadde bwolina?Ye……………Nedda……………..

Obba ye, buyitibwa butya................................................................................................

1. Omannyi kiki ekya buletea?Ye………… Nedda……………

Oba ye , kiki ekya buleeta?

………………………………………………………………………………………………………………………………………………………………………………………………

Wali obaddeko nomulwadde wa kafubba…………………………………………………..

Oba ye emyakkagringa emmeka?..........................................................................................

1. **Okukozesa ebilagalalagala**

Ofuweta segereti**?**………………………………………………………………………

1. Ate omwenge?………………………………………………………………………
2. Waliwo ekirara kyonna kyo kozesa? …………………………………………………

Bwoba okilina kinnyonnyole…………………………………………………………

1. **Eby’eddwaliro**

Okugemebwa kwa BCG…………………………………………………………….

Obulwaddebwa sukaali………...................................................................................

kookolo ………………………………………………………………………………..

Obuzito…………………………………………………………………………………

Okukolola………………………………………………………………………………

Obuwuka obuleeta ssirimu…………………………….…………………………

Ekifannayi kyo mukifuba ……………………………………………..........

Okuzulibwa mu obuwuka obuleta akafuba …………………………………………

**EBIVA MUMUSAYI**

Glucose levels........................................... mmol/l

Vitamin D levels……………………..…. ng/ml

Cathelicidin levels………………………ng/ml

Cytokine levels…………………………..pg/ml

Biochemical tests

**..................................................................................................................**

**..................................................................................................................**

**...................................................................................................................**

**..................................................................................................................**

**GC ISOFORMS**

*Gc1F* …………………………………………%

*Gc1S*…………………………………………%

*Gc 2*…………………………………………. %
